# Effect of Extracellular Vesicles From Multiple Cells on Vascular Smooth Muscle Cells in Atherosclerosis

**DOI:** 10.3389/fphar.2022.857331

**Published:** 2022-05-10

**Authors:** Tong Li, Baofu Wang, Hao Ding, Shiqi Chen, Weiting Cheng, Yang Li, Xiaoxiao Wu, Lei Wang, Yangyang Jiang, Ziwen Lu, Yu Teng, Sha Su, Xiaowan Han, Mingjing Zhao

**Affiliations:** ^1^ Key Laboratory of Chinese Internal Medicine of Ministry of Education, Dongzhimen Hospital, Beijing University of Chinese Medicine, Beijing, China; ^2^ Department of Oncology, Shanxi Traditional Chinese Medical Hospital, Taiyuan, China; ^3^ Department of Cardiac Rehabilitation, Dongzhimen Hospital, Beijing University of Chinese Medicine, Beijing, China

**Keywords:** atherosclerosis, endothelial cells, extracellular vesicles, macrophages, vascular smooth muscle cells

## Abstract

Atherosclerosis (AS)-related diseases are still the main cause of death in clinical patients. The phenotype switching, proliferation, migration, and secretion of vascular smooth muscle cells (VSMCs) have a pivotal role in atherosclerosis. Although numerous research studies have elucidated the role of VSMCs in AS, their potential functional regulations continue to be explored. The formation of AS involves various cells, such as endothelial cells, smooth muscle cells, and macrophages. Therefore, intercellular communication of blood vessels cannot be ignored due to closely connected endothelia, media, and adventitia. Extracellular vesicles (EVs), as the vectors of cell-to-cell communication, can deliver proteins and nucleic acids of parent cells to the recipient cells. EVs have emerged as being central in intercellular communication and play a vital role in the pathophysiologic mechanisms of AS. This review summarizes the effects of extracellular vesicles (EVs) derived from multiple cells (endothelial cells, macrophages, mesenchymal stem cells, etc.) on VSMCs in AS. The key findings of this review are as follows: 1) endothelial cell–derived EVs (EEVs) have anti- or pro-atherogenic effects on VSMCs; 2) macrophage-derived EVs (MEVs) aggravate the proliferation and migration of VSMCs; 3) mesenchymal stem cells can inhibit VSMCs; and 4) the proliferation and migration of VSMCs can be inhibited by the treatment of EVs with atherosclerosis-protective factors and promoted by noxious stimulants. These results suggested that EVs have the same functional properties as treated parent cells, which might provide vital guidance for treating AS.

## Introduction

Atherosclerosis (AS) is a chronic inflammation of the vascular system caused by the interaction of endothelial dysfunction, lipid metabolism disorder, and infiltration of inflammatory cells (Li et al., 2018). Vascular smooth muscle cells (VSMCs) are major cells in the media layer of arteries, critical for maintaining the integrity of the arterial wall. Under physiological conditions, VSMCs exhibit low proliferation and synthesis. The cellular function and phenotype can be regulated by cytokines and hemorheology (Wan et al., 2019; Ledard et al., 2020). The proliferation and migration of VSMCs and secretion of extracellular matrix (ECM) are critical steps in the occurrence and development of AS. In early AS, VSMCs transform from a contractile to synthetic phenotype and phagocytose lipids and then transform into foam cells, thus participating in the formation of lipid pools. VSMCs then proliferate and secrete ECM, resulting in the thickening of the pathological intima while preventing the rupture of fiber caps and stabilizing plaques in advanced AS (Basatemur et al., 2019). In addition, a series of cells, such as adventitial fibroblasts, endothelial cells (ECs), and macrophages act on VSMCs, affecting their proliferation, migration, and apoptosis, thus regulating the formation and development of AS.

Extracellular vesicles (EVs) have a significant mediating role in regulating vascular function and are closely related to the occurrence and development of cardiovascular diseases (Hulsmans and Holvoet, 2013). EVs contain proteins, RNAs, and lipids and represent an astonishing tool for transferring biochemical properties from cell to cell (Charla et al., 2020). EVs carry molecular signatures of both health and disease and are thus considered indicators of diagnosis and prognosis, and sometimes as a vector of AS-targeted therapy. Over the years, the application of EVs in the diagnosis, prognosis, and treatment of AS has been investigated. This review summarizes the effects of EVs from multiple cells (endothelial cells, macrophages, mesenchymal stem cells, etc.) on VSMCs in AS.

## Role of Vascular Smooth Muscle Cells in Atherosclerosis

### Vascular Smooth Muscle Cells’ Phenotypic Switching

Normally, VSMCs are in a contractile phenotype. When stimulated, VSMCs dedifferentiate to a synthetic state characterized by decreased myofilament density and contractile protein expression. During this phase, the expression of the contractile protein, alpha smooth muscle actin (α-SMA), and smooth muscle 22α decreases, while the expression of synthetic markers osteopontin and retinol-binding protein increases (Lacolley et al., 2017; Lu et al., 2018). Synthetic VSMCs show increased proliferation and migration ability, which are accompanied by secretion of ECM, matrix metalloproteinases (MMPs), pro-inflammatory cytokines, and exosomes. Exosomes trigger the differentiation of adjacent VSMCs into osteochondral VSMCs, which are characterized by runt-related transcription factor 2 and osteopontin expression, calcium deposition release, and calcification vesicles (Kapustin and Shanahan, 2016; Durham et al., 2018).

VSMC-derived intermediate cells, termed “SEM” cells, are pluripotent and can differentiate into macrophage-like and fibrochondrocyte-like cells (Pan et al., 2020). Macrophage colony–stimulating factors can induce the transformation of SEM cells into CD68^+^ macrophages, while at the same time, very few CD68^+^ cells were found induced by non-SEM cells (Manzanero, 2012). A previous study also showed that the level of various fibroblast markers, such as collagen type I, fibronectin, fibroblast-specific protein 1, and vimentin, is remarkably increased in SEM cells treated with connective tissue growth factor (Lee et al., 2010). Moreover, VSMCs’ transition to SEM cells is reversible. The marker of VSMCs' actin alpha 2 (ACTA2) is infrequent in SEM and non-SEM cells (mainly VSMC-derived fibrochondrocyte), yet a higher percentage of ACTA2+ cells was found in SEM cells than in non-SEM cells after induction of the transforming growth factor β1 (TGF-β1, VSMCs’ differentiation promoter) for 3 days (Pan et al., 2020).

### Vascular Smooth Muscle Cells’ Proliferation and Migration

Accumulation of VSMCs is a marker of atherosclerosis and vascular injury. In the past, it was believed that AS was the involvement of media VSMCs after endothelial injury and that the continuous proliferation of VSMCs was accumulated by lesion injury or inflammation. Now, the proliferation of VSMCs or cells derived from advanced atherosclerotic plaques is found to be low. Recent lineage-tracing studies have suggested that VSMCs’ proliferation begin in the media, after which the cells migrate to the intima, where they continue to divide in the oligoclonal mode (Chappell et al., 2016). The cells proliferate to form fibrous caps and then invade the plaque core (Misra et al., 2018); VSMCs in injury-induced neointimal lesions and atherosclerotic plaques are oligoclonal derived from a few dilated cells. Lineage tracing also indicates that a single VSMC contributes to the formation of α-SMA–positive fibrous cap and Mac3-expressing macrophage-like plaque core cells. The co-staining of phenotypic markers further identifies the double-positive α-SMA^+^ Mac3^+^ cell population, specific to the VSMC-derived plaque cells. On the contrary, VSMC-derived cells producing neointima after a vascular injury usually retain the expression of VSMC markers, and the upregulation of Mac3 in these cells is not obvious. It has also been demonstrated that the extensive proliferation of a low proportion of highly plastic VSMCs leads to the accumulation of VSMCs after injury and in atherosclerotic plaques. Thus, therapeutic targeting of these hyper-proliferative VSMCs may effectively reduce vascular diseases without affecting the vascular integrity.

External factors participate in regulating cells proliferation. Noncoding RNA can interact with proteins, DNA, and RNA to participate in VSMCs’ proliferation. The expression of miR143/145 decreases in atherosclerotic vascular cells and can block VSMC de-differentiation and proliferation by inhibiting KLF4 and Elk1 through binding with their mRNA 3′UTR region (Cordes et al., 2009). Mahmoud et al. (2019) suggested that long noncoding RNA (LncRNA) SMILR promotes VSMCs’ proliferation by directly regulating mitosis, and its expression is increased in stable and unstable atherosclerotic plaques. Moreover, LncRNA MALAT1 stimulates proliferation and migration of VSMCs and promotes aortic stiffness (Song et al., 2018; Yu et al., 2018).

VSMCs generally migrate to the intima and proliferate to form fibrous caps (Allahverdian et al., 2018). Migration of VSMCs in the media may be preceded by both mitotic and non-mitotic VSMCs, which promote the formation of lesions (Webster et al., 1974; Clowes and Schwartz, 1985). However, the lineage-tracing study showed that VSMCs’ migration was independent of proliferation and was not a major factor in the pathogenesis of the disease. Similarly, neointimal plaques derived from VSMCs were observed to connect with media plaques expressing the same color, suggesting that VSMCs proliferate in the media and thus predate migration (Chappell et al., 2016).

miRNA also has an important role in the migration of VSMCs. Studies have shown that miRNA-26a, miRNA-181b, miRNA-135b-5p, and miRNA-499a-3p promote the migration of VSMCs, while miRNA-599 and miRNA-132 have a negative effect (Gao et al., 2016). For example, miRNA-181b can promote proliferation and migration of VSMCs by activating phosphatidylinositol kinase-3(PI3K)/mitogen-activated protein kinase (MAPK) (Li et al., 2015), while miRNA-599 inhibits VSMCs migration by targeting TGF-β2 mRNA, thereby decreasing the expression of proliferating nuclear antigen (Xie et al., 2015).

### Vascular Smooth Muscle Cells’ Secretion

VSMCs secrete various biologically active molecules, namely, matrix proteins and pro-inflammatory mediators, some of which are encapsulated in vesicles that are released from the cell surface and transmit signals between cells. ECM produced by VSMCs is the main structural component of the vascular wall. The interaction between the two is a dynamic bidirectional process, and the content of the ECM depends on the balance of production and degradation (Barallobre-Barreiro et al., 2020).

During early plaque formation, MMPs affect VSMCs’ migration by degrading the connective tissue structure around VSMCs (Johnson, 2017). A variety of matrix-degrading enzymes are secreted by synthetic VSMCs that can lead to the death of the neighboring cells (Johnson, 2017; Allahverdian et al., 2018). Pro-inflammatory cytokines interleukin-1β (IL-1β), IL-6, and monocyte chemoattractant protein-1 (MCP-1) promote atherosclerosis by stimulating monocyte recruitment and cell death (Orr et al., 2010). Synthetic VSMCs express a series of adhesion molecules and toll-like receptors that promote monocyte recruitment and regulate intracellular inflammatory signals.

In addition, VSMCs secrete EVs, which contain phosphatidylserine PS, annexin A6, and a low concentration of calcification inhibitors that may lead to vascular calcification (Kapustin et al., 2015). Proudfoot et al. (2000) suggested that matrix vesicles containing apoptotic VSMC remnants can serve as nucleation sites for plaques calcification. In addition, Schurgers et al. (2018) found that osteochondrocyte-like VSMCs secreted calcified vesicles which can promote calcification. Senescent cells released more EVs than non-senescent cells, promoting cell proliferation, inflammatory response, wound healing, and DNA damage (Borghesan et al., 2019; Basisty et al., 2020).

## Role of Extracellular Vesicles–Regulated Vascular Smooth Muscle Cells in Atherosclerosis

### Characterization of Extracellular Vesicles

EVs are membrane-bound phospholipid vesicles secreted by cells. EVs carry proteins, nucleic acids, and other substances transmitted between cells and have a critical role in regulating cell homeostasis and pathological development (Colombo et al., 2014; Lo Cicero et al., 2015). According to biogenesis, origin, and size, EVs can be classified into exosomes (40–200 nm), microvesicles (MVs) and microparticles (200–2000 nm in size), and apoptotic bodies (500–2000 nm) (Shao et al., 2018; van Niel et al., 2018). The production of exosomes can be divided into three steps: firstly, the endosome is formed by the inward budding of the cellular plasma membrane. Further inward budding of the endosome then leads to the formation of a multivesicular body (Piper and Katzmann, 2007). Finally, the multivesicular body fuses with the plasma membrane, releasing the vesicles (Théry, 2011). MVs are produced by outward budding and division of the plasma membrane (Raposo and Stoorvogel, 2013).

The lipid distribution of the membrane bilayer is asymmetrical. The outer layer is enriched with phosphatidylcholine and sphingomyelin, while the inner layer is predominantly composed of phosphatidylserine and phosphatidylethanolamine (Zwaal and Schroit, 1997). The influx of cytoplasmic Ca2^+^ can disrupt this asymmetry by activating enzymes that facilitate the mixing of transport lipids. This activation leads to a redistribution of phospholipid bilayers across the membrane, promoting membrane blistering. Ca2^+^-dependent proteolysis simultaneously degrades membrane-associated cytoskeleton, accelerating the budding process (Hugel et al., 2005). High-speed centrifugation (<100000*g*) and flow cytometry are used to extract and detect specific MVs of different cell origins. Exosomes are commonly isolated by ultracentrifugation or by using commercial kits. The morphology of exosomes is then examined by transmission electron microscopy, while the size is evaluated by nanoparticle tracking analysis. EV-associated proteins, such as tetraspanin proteins (namely, CD9, CD63, and CD81), are detected by western blotting.

Exosomes and MVs contain nucleic acids, namely, miRNAs, mRNA (Valadi et al., 2007; Skog et al., 2008), DNA (Balaj et al., 2011; Thakur et al., 2014), and other noncoding RNAs. The use of EVs’ RNA as diagnostic biomarkers has become a hot research topic in recent years. EVs have already been used as biomarkers for autoimmune and circulatory diseases and cancer (Happel et al., 2020; Xu et al., 2020).

### Extracellular Vesicles–Mediated Crosstalk Between Multiples Cells and Vascular Smooth Muscle Cells in Atherosclerosis

**FIGURE 1:**
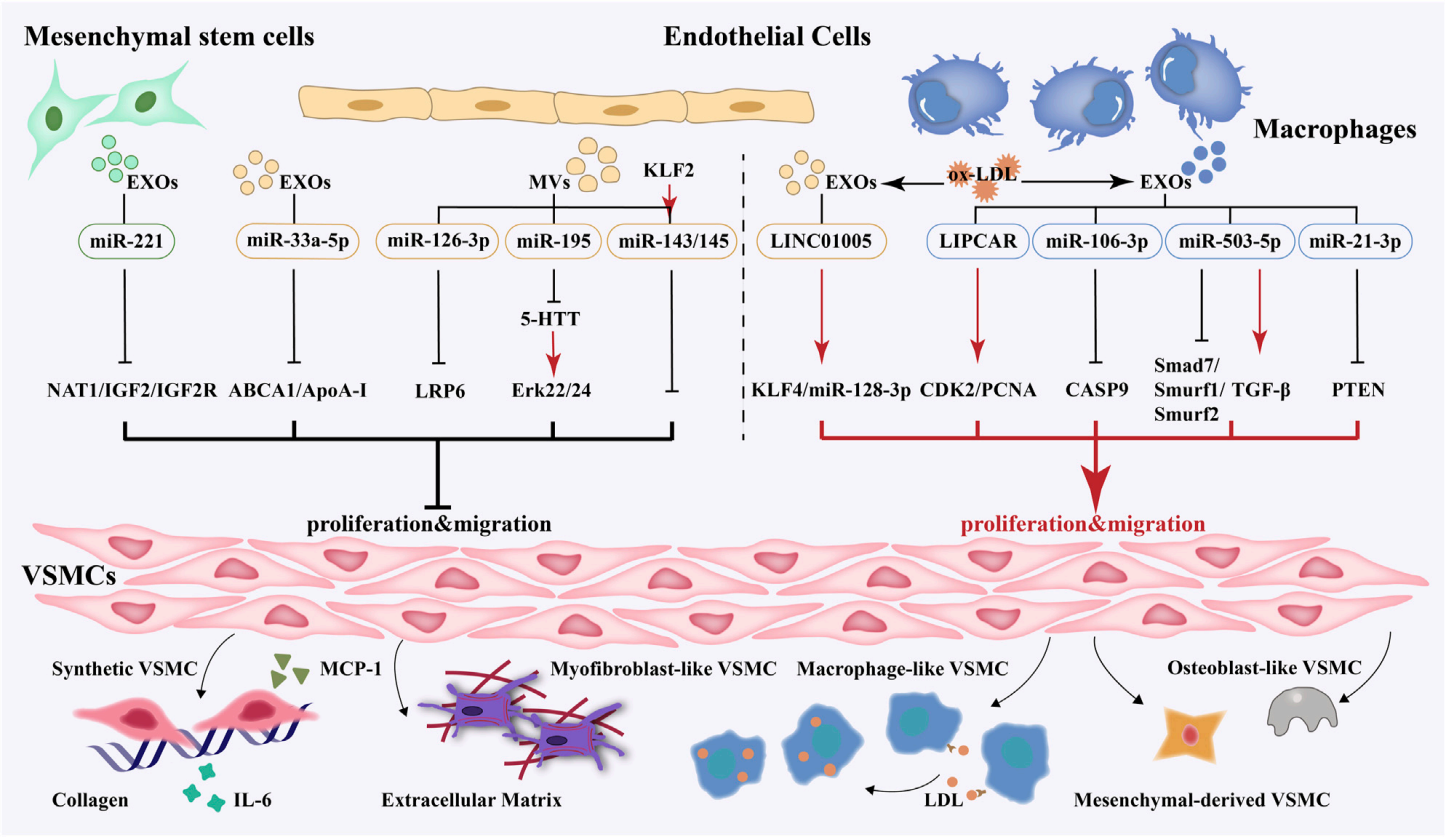
EVs-mediated crosstalk between multiple cells and VSMCs and phenotype switching, proliferation, migration and secretion of VSMCs.

Intercellular communication is a vital part of regulating vascular function. EVs mediate the communication between cells during the development of atherosclerosis and play a role in delivering proteins, nucleic acids, or other active substances to the receptor cells. Through a comprehensive literature search, 16 studies were extracted to summarize the effect of extracellular vesicles derived from multiple cells on smooth muscle cells. EVs derived from endothelial cells, macrophages, and mesenchymal stem cells exert various effects on VSMCs’ proliferation and migration. Krüppel-like factor 2 (KLF2), 5-hydroxytryptamine transporter (5-HTT) inhibitor, miR-33a-5p antagomir, and miR-221 agomir are the protective factors of atherosclerosis. After being treated with the above-mentioned factors, EVs could inhibit the proliferation and migration of VSMCs. However, EVs produced by pro-atherogenic factors, such as oxidized low-density lipoprotein (ox-LDL) and nicotine, promoted the proliferation and migration of VSMCs. More in-depth studies have found that miR-33a-5p, miR-126, and miR-221 carried by the vesicles had a protective effect on VSMCs, while others, such as miR-128-3p, miR-21, miR-106, miR-503-5p, and LIPCAR showed detrimental effects. More study details are given in Table 1 and Figure 1.

**TABLE 1:** EVs-mediated crosstalk between multiple cells and VSMCs in AS.

	Vesicular origin	Type	Stimulants	Cargo mediators	Target pathway	Functions	References
Anti-atherogenic↓	ECs	MVs	KLF2	miR-143/145	KLF2/miR-143/145	Prevented VSMCs’ de-differentiation, limited the progression of atherosclerosis	Hergenreider et al. (2012)
		MVs	5-HTT inhibitors	miR-195	miR-195/5-HTT/Erk22/24	Inhibited VSMCs’ proliferation and migration	Gu et al. (2017)
		MVs	miR-126 mimic and inhibitor	miR-126-3p	miR-126-3p/LRP6	Inhibited proliferation and migration of VSMCs and neointima formation	Jansen et al. (2017)
		Exosomes	miR-33a-5p antagomir	miR-33a-5p	miR-33a-5p/ABCA1/ApoA-I	Increased ABCA1 expression, enhanced ApoA-I–mediated cholesterol efflux, inhibited the development of AS	Stamatikos et al. (2020)
		Exosomes	-	-	-	Reduced VSMCs’ proliferation and migration and lipid accumulation	Xiang et al. (2021)
	MSCs	Exosomes	miR-221 agomir	miRNA-221	miRNA-221/NAT1/IGF2/IGF2R	Suppressed atherosclerotic plaque formation	Guo et al. (2020)
	Adipose MSCs	Exosomes	-	-	MAPK/Akt	Inhibited proliferation and migration of VSMCs	Liu et al. (2016a)

Pro-atherogenic ↑	EPCs	Exosomes	-	-	-	Promoted VSMCs’ proliferation and migration	Kong et al. (2018)
	ECs	Exosomes	CD137	-	TET2/CD137/PDGF-BB	Promoted phenotypic switching of VSMCs and neointimal formation	Li et al. (2020)
		Exosomes	ox-LDL	LINC01005	LINC01005/KLF4/miR-128-3p	Promoted VSMCs’ phenotype switch, proliferation, and migration	Zhang et al. (2020)
	Macrophages	EVs	Western diet	-	-	Promoted VSMCs’ proliferation	Wang et al. (2018a)
		Exosomes	ox-LDL	-	Erk/Akt	Promoted adhesion and migration of VSMCs	Niu et al. (2016)
		Exosomes	Nicotine	miR-21-3p	miR-21-3p/PTEN	Promoted proliferation and migration of VSMCs	Zhu et al. (2019)
		Exosomes	ox-LDL	miR-106-3p	miR-106-3p/CASP9	Promoted proliferation and migration of VSMCs	Liu et al. (2020)
		Exosomes	ox-LDL	miRNA-503-5p	miRNA-503-5p/Smad7/Smurf1/Smurf2/TGF-β	Promoted proliferation and migration of VSMCs	Wang et al. (2021a)
		Exosomes	ox-LDL	LIPCAR	LIPCAR/CDK2/PCNA	Promoted proliferation and migration of VSMCs	Hu et al. (2021)

5-HTT, 5-hydroxytryptamine transporter; VSMCs, vascular smooth muscle cells; ECs, endothelial cells; EPCs, endothelial progenitor cells; KLF2, Krüppel-like factor 2; LRP6, lipoprotein receptor–related protein 6; MSCs, mesenchymal stem cells; MVs, microvesicles; ox-LDL, oxidized low-density lipoprotein.

### Effect of Endothelial Cell–Derived Extracellular Vesicles on Vascular Smooth Muscle Cells in Atherosclerosis

EEVs can regulate endothelial barrier function, control VSMCs' phenotype, modulate monocytes activation, and affect atherosclerotic lesion formation in AS (Njock et al., 2015; Zheng et al., 2017; Wang et al., 2021). The blood vessels are mainly composed of ECs and VSMCs; the interaction between the two is essential for the repair and remodeling of blood vessel growth (Li et al., 2018). Activation, proliferation, and migration of VSMCs can promote the formation of atherosclerotic plaques. VSMCs' phenotype is regulated by several environmental factors, such as growth factors, cytokines, and injury stimulation (Owens et al., 2004; Davis-Dusenbery et al., 2011). As a novel intercellular communication vector, EVs have received extensive attention. Evidence has shown that EEVs may have both anti- or pro-atherogenic effects on VSMCs.

The protective effects are mainly associated with miRNA contained in EVs. Boon and Horrevoets (2009) indicated that KLF2 has an important role in anti-atherosclerosis by regulating endothelial biological activity, mediating atherosclerosis induced by shear stress and protecting the endothelial phenotype. KLF2 binds to miR-143/145 and induces an increment of the cluster which regulates the phenotype of VSMCs. EVs released by KLF2-stimulated HUVECs were enriched in miR-143/145, and the expression of miR-143/145–targeted genes declined in coculture VSMCs. When miR-143/145–deficient ECs are cocultured with VSMCs, the miRNA targets were suppressed in VSMCs. KLF2 conversion led to a 30-fold enrichment of miR-143/145 in EVs, while the exosomes-depleted supernatant did not show an upregulation of the miR-143/145 levels. In addition, EVs produced by the endothelial cells expressing KLF2 also reduced the formation of atherosclerotic aortic lesions in ApoE^-/-^ mice. The results suggested that KLF2 mediates miR-143/145 transferred from endothelial cells to VSMCs in EVs to maintain the differentiation status of VSMCs and atheroprotective effects (Hergenreider et al., 2012).

5-hydroxytryptamine is an important bioactive substance in the body, which promotes the formation of macrophage-derived foam cells. It can also promote the proliferation and migration of VSMCs through LDL and ox-LDL (Koba et al., 1999), as well as 5-hydroxytryptamine transporter (5-HTT) (Wang et al., 2015). The level of 5-HTT increased in the injured carotid artery and the overexpression of 5-HTT–induced VSMCs’ proliferation. ECs’ conditional medium (EC-CM) hampered the proliferation and expression of 5-HTT in SMCs. After ECs’ transfection with miR-195 inhibitors, EC-CM was added to culture VSMCs and the expression of 5-HTT did not decline in them. These results showed that EEVs transforming miR-195 to VSMCs restrained the expression of 5-HTT, thereby inhibiting the proliferation of VSMCs by enhancing Erk42/44 phosphorylation level (Gu et al., 2017).

Lipid accumulation of intimal macrophages and VSMCs are also an essential driving factor for AS (Tabas et al., 2015). ATP-binding cassette transporter A1 (ABCA1) can transport intracellular cholesterol to ApoA-I, forming a high-density lipoprotein precursor, such that excess cholesterol can be transported to the liver for reuse after metabolism or excretion, thus reducing the formation of foam cells and inhibiting the occurrence and development of AS (Qian et al., 2017). Cholesterol accumulation in VSMCs induces cell differentiation into foam cell phenotype. In addition, cholesterol deposition in VSMCs downregulates the expression of VSMC markers ACTA2 and calmodulin and increases the expression of inflammation-related genes. Endothelial cells release exosomes containing miR-33a-5p, a microRNA that restrained cholesterol efflux by silencing ABCA1. Stamatikos et al. (2020) transfected ECs with anti-miR-33a-5p, which was then incubated with macrophages or VSMCs. Exosome-mediated transfer of anti-miR-33a-5p increased ABCA1 expression and enhanced ApoA-I–mediated cholesterol efflux, inhibiting the development of AS. However, the effects were not observed when exosomes were removed from the medium. Furthermore, EEVs absorbed by VSMCs suppressed the proliferation, migration, and lipid deposition of VSMCs, while LPS-induced EEVs promoted the proliferation of VSMCs. Also, GW4689, an inhibitor of EVs, prevented the effect of EEVs on the proliferation and migration of VSMCs (Xiang et al., 2021).

Injections with endothelial microparticles (EMPs) reduced neointima formation in mice after vascular injury. Low-density lipoprotein receptor–related protein 6 (LRP6), a target of miR-126, is involved in regulating the proliferation of VSMCs and neointima formation. Upregulation of miR-126 in EMPs can reduce LRP expression, thereby inhibiting the proliferation and migration of VSMCs and neointima formation. The results indicated that EMPs delivered miR-126-3p to VSMCs and inhibited the expression of LRP6, thus reducing VSMCs’ proliferation and disrupting neointima formation and vascular remodeling (Jansen et al., 2017).

Recent studies have shown that endothelial progenitor cells (EPCs) do not directly differentiate into mature ECs but utilize paracrine mechanisms through which they potentially participate in enhancing re-endothelialization (Hagensen et al., 2010, 2012). EPC-derived exosomes were injected into rats to investigate whether they could regulate re-endothelialization. It was found that the re-endothelialization area of the exosomes group was bigger than that of the control group, and both the intimal-to-medial area ratio and VSMCs proliferation in the exosomes group were markedly decreased when compared with those in the control group. At the same time, Kong et al. (2018) found that the exosomes promoted VSMCs' proliferation and migration *in vitro*.

EEVs promote atherosclerosis. TET2 is expressed in endothelial cells and can protect cells against inflammation. It is also regarded as a regulator of the transition to the VSMCs' phenotype, and its reduction leads to VSMCs' de-differentiation. The activation of CD137 signaling in ECs has a key role in inducing the immune and inflammatory response of AS. Injection with EC-derived exosomes significantly declined the intima/media ratio and neointima area, whereas CD137L (CD137 ligand) reversed the effect. Exosomes derived from ECs decreased the migration of PDGF-BB–induced VSMCs; however, the endothelial CD137 pathway was activated during this process, and the TET2 content of the endothelial-derived exosomes was repressed, promoting the phenotype switch and migration of VSMCs. Overexpression of TET2 in exosomes weakened the CD137 signaling–stimulated pro-phenotypic switch of VSMCs *in vitro* and *in vivo*, thus eventually attenuating plaque formation and AS development (Li et al., 2020).

LncRNA, a type of noncoding RNA, regulates gene expression at the transcriptional, posttranscriptional, and epigenetic levels (Kwok and Tay, 2017). It can be transferred from EVs of parent to recipient cells. Exosomal LINC01005 from ECs treated with ox-LDL promoted VSMCs’ phenotype switch, proliferation, and migration by enhancing KLF4 expression *via* competitively binding to miR-128-3p. Of note, the effects were negated by upregulation of miR-128-3p *via* miR-128-3p mimic and silencing of KLF4 (Zhang et al., 2020).

### Effect of Macrophage-Derived Extracellular Vesicles on Vascular Smooth Muscle Cells in Atherosclerosis

MEVs can induce macrophages polarization, modulate proliferation and migration of VSMCs, and regulate inflammatory response and lipid deposition in AS (Nguyen et al., 2018; Zhang et al., 2019; Bouchareychas et al., 2020). Macrophages and VSMCs have a critical role in plaque necrosis and rupture. Macrophages can secrete pro-inflammatory factors to maintain local inflammation in plaque. At the same time, they interact with T cells and VSMCs to enhance inflammation and promote lipoprotein retention (Moore et al., 2013). The transformation of VSMCs into the macrophage phenotype may be driven by lipid accumulation due to the cholesterol load in the culture (Rong et al., 2003), and also reversed by stimulating cholesterol efflux through ApoA-I and high-density lipoprotein (Allahverdian et al., 2014). Previous studies have suggested that MEVs promote smooth muscle cell proliferation and migration, thereby contributing to the development of atherosclerosis.

Four out of six studies made use of involved ox-LDL to treat macrophages (Niu et al., 2016; Liu et al., 2020; Hu et al., 2021; Wang et al., 2021a). Ox-LDL promotes the migration and proliferation of VSMCs by activating MAPK and other signaling pathways, upregulating the expression of adhesion molecules, inflammatory factors, and chemokines (Liu et al., 2014). Yet, high concentrations of ox-LDL can induce apoptosis of VSMCs, resulting in decreased plaque stability and easy rupture (Obermayer et al., 2018). In addition, ox-LDL stimulates vascular endothelial cells to express chemokines that induce monocytes to adhere to the vascular endothelium and move to the subintimal layer. The monocytes then differentiate into macrophages, which engulf ox-LDL receptors to form foam cells (Chistiakov et al., 2019).

Niu et al. (2016) found a higher level of leukocyte-derived EVs in patients with atherosclerosis than in healthy subjects. These EVs accelerated the migration and adhesion of VSMCs. Moreover, *in vitro* experiments suggested that foam cells produced more EVs than normal macrophages. In addition, proteomic results suggested that foam cell–derived EVs might promote adhesion and migration of VSMCs by regulating the actin skeleton and local adhesion pathways. Further validation revealed that foam cell–derived EVs may activate ERK and Akt pathway proteins.

In ox-LDL–treated macrophages, miR-106a-3p was significantly enriched in the exosomes, which were absorbed by VSMCs, causing a reduction in its target gene CASP9. miR-106a-3p overexpression and exosomes knockdown promoted and repressed proliferation and migration of VSMCs, respectively. This research revealed that exosomal miR-106a–mediated macrophage–VSMC crosstalk promoted VSMC proliferation and suppressed apoptosis *via* inhibition of CASP9 expression, thus further promoting the development of atherosclerosis (Liu et al., 2020).

Wang et al. (2021) found that EVs released by ox-LDL–treated macrophages containing miRNA-503-5p increased the proliferation and migration of VSMCs, while downregulation miR-503-5p attenuated these effects. Also, proliferation and migration of VSMCs were accelerated by downregulating the expressions of Smad7, Smurf1, and Smurf2 and elevating TGF-β, then exacerbating AS.

LncRNA LIPCAR participates in the development of AS, while excessive expression of LIPCAR significantly promotes phenotype switching, proliferation, and migration of VSMCs (Wang et al., 2019). The level of LIPCAR increased in exosomes from human myeloid leukemia mononuclear cells (THP-1) which was treated with ox-LDL. Furthermore, Hu et al. (2021) suggested that exosomes accelerated the proliferation and migration of VSMCs by upregulating CDK2 and PCNA, while this effect could be reversed by LIPCAR.

EVs stimulated by smoke and hyperlipidemia, risk factors of AS, display pro-atherogenic effects. Cigarette smoke is one of the risk factors of atherosclerosis (Messner and Bernhard, 2014). Wang et al. (2019) suggested that nicotine, a major component of cigarettes, not only directly activated the migration and proliferation of plaque cells but also enhanced the pro-inflammatory communication between macrophages and VSMCs, thereby promoting the occurrence of AS. Nicotine stimulated macrophages to produce exosomes, enriched with miR-21-3p, which were reported to join in vascular injury and repair (Liu et al., 2016). In a previous study, VSMCs were transfected with miR-21-3p mimics and miR-21-3p inhibitors and then incubated with EVs. After miR-21-3p mimic transfection, the migration and proliferation of VSMCs were obviously increased. The target gene of miR-21, phosphate and tensin homolog (PTEN), was selectively knocked down and the increment of the migration and proliferation of VSMCs emerged. The expression of PTEN was inhibited and VSMCs’ proliferation and migration were enhanced by EVs-treated VSMCs, which exacerbated atherosclerosis progression (Zhu et al., 2019).

EVs derived from macrophage foam cells, which were isolated from mice fed on Western diet, promoted VSMCs proliferation. However, the exact mechanism is unclear (Wang et al., 2018).

### Effect of Mesenchymal Stem Cell–Derived Extracellular Vesicles on Vascular Smooth Muscle Cells in Atherosclerosis

Mesenchymal stem cells (MSCs) are considered pluripotent stem cells with great therapeutic potential. MSCs replace damaged tissue by differentiating into various cell lineages, regulate immune response, and secrete EVs by paracrine function. Mesenchymal stem cells–extracellular vesicles (MSCs-EVs) harbor anti-atherogenic effects, such as inhibiting intimal hyperplasia, suppressing inflammation, and promoting M2 macrophage polarization (Chen et al., 2016; Li et al., 2019). Two studies of MSCs-EVs have shown a protective effect on VSMCs. Adipose mesenchymal stem cells–derived EVs inhibited the proliferation and migration of VSMCs. The expression of IL-6 and MCP-1 and the phosphorylation of MAPK and Akt declined after treatment with EVs.

The involvement of pro-inflammatory cytokines might promote the proliferation and migration of VSMCs (Liu et al., 2016). miR-221 is downregulated in patients with AS and in AS plaques (Tsai et al., 2013), and the lack of miR-221 enhances plaque instability and rupture (Bazan et al., 2015). Simultaneously, elevated miR-221 may stabilize vulnerable atherosclerotic plaques by inhibiting inflammation (Ye et al., 2018). Transmission of miR-221 from EVs derived from MSCs can inhibit lipid deposition and atherosclerotic plaque formation. EVs with high miR-221 expression increased miR-221 in the aorta and reduced NAT1 and atherosclerotic plaque formation in ApoE^-/-^ mice. MSCs-EVs, including miR-221, were absorbed by ox-LDL–treated VSMCs and decreased the target gene NAT1, thereby suppressing the activation of the IGF2/IGF2R signaling pathway to inhibit atherosclerotic plaque formation (Guo et al., 2020).

## Effect of Multiple Extracellular Vesicles on Nonvascular Smooth Muscle Cells in Atherosclerosis

According to the existing literature, EVs have been proven to possess anti- or pro-atherogenic effects. EVs could regulate vascular inflammation, cholesterol metabolism, angiogenesis, plaque stability, and thrombosis through intercellular communication.

Monocytes and macrophages are important cell types those participate in atherosclerotic inflammation progression. The monocyte-derived EVs (MoEVs) isolated from human atherosclerotic plaques increase intercellular cell adhesion molecule-1 (ICAM-1), vascular cell adhesion molecule, and E-selectin, leading to increased leukocyte adhesion and transmigration (Rautou et al., 2011). In addition, MoEVs induce endothelial cells and leukocytes to release pro-inflammatory cytokines, in particular IL-6 and IL-8, which in turn promote the adhesion of cells (Boulanger et al., 2017). Hoyer et al. (2012) found mounting monocyte and T-cell infiltrated into the vessel wall, and enhanced plaque formation in ApoE^-/-^ mice treated with MoEVs. *In vitro* study showed MoEVs increased the generation of pro-inflammation factors of chemokine receptor 2, intracellular reactive oxygen species, IL-6, and ICAM-1. EVs are capable of enhancing immunomodulatory responses and diminishing pro-inflammatory responses. EEVs transfer miR-10a to the monocyte by targeting the inflammatory pathway of NF-κB/MAP3K7/IRAK4 to repress inflammatory signaling (Njock et al., 2015). In addition, EEVs reduce the M1 macrophage phenotype with a transition to the M2 anti-inflammatory macrophage phenotype and can be absorbed by the neighboring ECs and transferred to recipient cells through functional miR-222, promoting anti-inflammatory effects by decreasing ICAM-1 expression (Deng et al., 2019).

The dynamic balance between cholesterol uptake, synthesis, and efflux regulates cholesterol homeostasis in macrophages. This process is closely regulated by EVs-mediated cellular interaction. Cholesterol efflux can reduce intracellular cholesterol accumulation, preventing the formation of foam cells and the occurrence of AS. Moreover, cholesterol efflux is correlated with miR-3129-5p of adipocyte-derived EVs. The more the adipocyte-derived EVs are released, the lower the cholesterol efflux from macrophages and ABCA1 is expressed (Barberio et al., 2019). CD4^+^-activated T lymphocytes infiltrate atherosclerotic plaques, induce T lymphocyte-releasing exosomes, and promote cholesterol accumulation and the expression of tumor necrosis factor-α (TNF-α) in THP-1, thereby facilitating AS (Zakharova et al., 2007). However, platelet-derived EVs (PEVs) exert anti-cholesterol aggregation effects and inhibit atherosclerotic thrombosis by suppressing ox-LDL binding and cholesterol accumulating in macrophages, affecting the class B scavenger receptor CD36 and inhibiting platelet thrombosis (Srikanthan et al., 2014).

The accumulation of EVs in atherosclerotic plaque indicates an endogenous signal of plaque neovascularization and vulnerability (Leroyer et al., 2008). EVs regulate angiogenesis and plaque stability, a major event in the switching from stable to unstable lesions. CD40^+^ EVs in atherosclerotic plaque stimulate endothelial proliferation and angiogenesis and may be involved in intra-plaque neovascularization. The CD40L-expressing EVs isolated from human atherosclerotic lesions stimulate endothelial cell proliferation and promote angiogenesis by involving vascular endothelial growth factor and PI3K/Akt following connection with endothelial CD40 (Leroyer et al., 2008). In addition, the transfer of microRNAs from EVs to recipient ECs can regulate angiogenesis. For example, under IL-3 stimulation, EVs secreted by ECs are transported to ECs’ recipients through miR-126-3p and pSTAT5 to induce angiogenesis (Lombardo et al., 2016). The promotion of angiogenesis in advanced plaques leads to instability and rupture of the plaque, thus accelerating the development of AS. Insulin-resistant adipocyte-derived exosomes can enter into HUVECs and atherosclerotic plaques, promote tube formation, increase vasa vasorum angiogenesis, the plaque burden, the vulnerability index, and the expression of angiogenesis-related factors (Wang et al., 2018).

Activated platelets releasing PEVs is an integral part of the thrombotic process. The procoagulant activity of platelet EVs in blood circulation is much higher than that of activated platelets (Sinauridze et al., 2007). PSGL-1 on PEVs activates platelets by binding to P-selectin in the endothelial injured area, which is conducive to thrombosis and atherosclerosis, and promotes the expansion of lesions (Suades et al., 2012). High levels of PEVs have been found in patients with coronary disease. Several studies have confirmed that increased PEV levels can enhance platelet and fibrin adhesion under high shear stress, injuring the atherosclerotic vessel wall (Suades et al., 2012; Mause, 2013). Tissue factor (TF) initiating coagulation is exposed to triggered thrombus formation (Biró et al., 2003). TF^+^ monocyte EVs, the second largest group of thrombogenic EVs, follow platelet EVs (Aharon et al., 2008). They are abundant in human atherosclerotic plaques and may be aggregated in the vascular injury site by combining with activated platelets (Del Conde et al., 2005; Furie and Furie, 2008).

## Role of Extracellular Vesicles as a Drug Vector

The use of endogenous exosomes as drug vectors has good biocompatibility and non-immunogenicity. It can improve the effective utilization rate and reduce the drug clearance rate. A recent study evaluated the anti-atherosclerotic effect of platelet-derived EV loaded with NLRP3 inhibitor MCC950. In ApoE-deficient mice, intravenous administration of PEVs mitigated inflammatory processes and atherosclerotic plaque formation, and inhibited macrophage and T-cell proliferation (Ma et al., 2021). Exosomes have also been used as drug vectors in the study of Chinese traditional medicine monomers. For example, exosomes loaded with curcumin increased the concentration and stability of curcumin *in vivo* and improved its therapeutic effect without obvious adverse reactions (Sun et al., 2010). However, the research on drug vectors of exosomes is still in its early phase, thus extensive research is still needed to optimize the targeting of exosomes as drug delivery vectors in the future.

## Conclusion

This review summarized the effect of multiple cells–derived EVs on VSMCs in atherosclerosis. Endothelial cell–derived EVs have dual effects on VSMCs, while macrophage-derived EVs can promote the proliferation and migration of VSMCs and impair AS. Moreover, studies on EVs derived from mesenchymal stem cells showed that these particular EVs have inhibiting effects on VSMCs. We also found that EVs containing miR-33a-5p, miR-126, and miR-221 were able to inhibit the proliferation and migration of VSMCs, displaying protective effects on AS, whereas miR-128-3p, miR-21, miR-106, miR-503-5p, and LIPCAR further aggravated the disease. Additionally, studies suggested that EVs derived from source cells treated with beneficial factors have an important role in anti-atherosclerosis, and the harmful stimulants promote the development of AS. Therefore, EVs used as drug vectors may be a novel approach in treating AS. This review also provides new insight into the complexity of VSMCs biology and the potential of cells as a target for therapeutic strategies in AS. Under physiological conditions, EVs mediate intercellular communication and are involved in maintaining homeostasis; under a pathological state, however, EVs are released by the parent cell and participate in the occurrence and development of the disease. The pro-atherogenic and anti-atherogenic EVs’ balance in different stages of atherosclerosis is still not very clear. Further studies are needed to verify whether the effect of EVs *in vivo* is consistent with that *in vitro*. Also, the methods for extraction and purification of EVs have not yet been unified. At this stage, EVs are still recommended only as auxiliary diagnostic indicators for a certain disease. Their limited clinical transformation and small numbers make them currently unavailable for the treatment of disease. Therefore, an in-depth study of EVs’ function and improvement in the rate of EVs’ acquisition is of great value for their clinical application.

## Abbreviations

5-HTT, 5-hydroxytryptamine transporter; α-SMA, smooth muscle acting; ABCA1, ATP-binding cassette transporter A1; ACTA2, actin alpha 2; AS, atherosclerosis; ECM, extracellular matrix; ECs, endothelial cells; EEVs, endothelial cell–derived EVs; EMPs, endothelial microparticles; EPCs, endothelial progenitor cells; EVs, extracellular vesicles; ICAM-1, intercellular cell adhesion molecule-1; IL-1β, interleukin-1β; KLF2, Krüppel-like factor 2; LncRNA, long noncoding RNA; LRP6, lipoprotein receptor–related protein 6; MAPK, mitogen-activated protein kinase; MCP-1, monocyte chemoattractant protein-1; MEVs, macrophage-derived EV; MoEVs, monocyte-derived EVs; MMPs matrix metalloproteinases.
